# A clinical and radiographic study of implants placed in autogenous bone grafts covered by either a platelet-rich fibrin membrane or deproteinised bovine bone mineral and a collagen membrane: a pilot randomised controlled clinical trial with a 2-year follow-up

**DOI:** 10.1186/s40729-021-00289-z

**Published:** 2021-02-08

**Authors:** Jens Hartlev, Søren Schou, Flemming Isidor, Sven Erik Nørholt

**Affiliations:** 1 Section for Oral Surgery and Oral Pathology, Department of Dentistry and Oral Health, Health, Aarhus University, Vennelyst Boulevard 9, DK-8000 Aarhus C, Denmark; 2 Department of Periodontology, School of Dentistry, Faculty of Health Sciences, University of Copenhagen, Noerre Alle 20, DK-2200 Copenhagen N, Denmark; 3 Section for Prosthetics, Department of Dentistry and Oral Health, Health, Aarhus University, Vennelyst Boulevard 9, DK-8000 Aarhus C, Denmark; 4 Department of Oral and Maxillofacial Surgery, Aarhus University Hospital, Palle Juul-Jensens Boulevard 99, DK-8200 Aarhus N, Denmark

**Keywords:** Dental implants, Follow-up study, Guided bone regeneration, Membrane, Platelet-rich fibrin, Ridge augmentation

## Abstract

**Purpose:**

To compare the survival and clinical performance of implants placed in sites previously augmented with autogenous bone grafts covered by either a platelet-rich fibrin (PRF) membrane (PRF group) or a standard procedure (gold standard) involving coverage of the autogenous bone graft with deproteinised bovine bone mineral and a resorbable collagen membrane (control group).

**Methods:**

A total of 27 partially edentulous patients (test *n* = 14, control *n* = 13) with indication for staged lateral bone block augmentation and dental implant placement were included. Twenty-four months after crown placement (range: 14–32 months), patients were recalled for a final clinical and radiographic follow-up. Outcome measures were implant survival, implant crown survival, clinical parameters of the implant, peri-implant marginal bone level, marginal bone level of adjacent tooth surfaces, biological and technical complications and patient-related outcome measures.

**Results:**

Two implants were lost in the control group (85% survival rate); none were lost in the PRF group (100% survival rate). None of the 26 initially placed implant crowns were lost, but one implant and therefore one implant crown were lost after 20 months. Consequently, the definitive implant crown survival was 92% (95% confidence interval (CI): 73–110%) in the control group and 100% in the PRF group. No statistical difference in implant survival rate (*p* = 0.13) or implant crown survival was seen between the groups (*p* = 0.28). The mean marginal bone level at the follow-up was 0.26 mm (95% CI: 0.01–0.50 mm) in the PRF group and 0.68 mm (95% CI: 0.41–0.96 mm) in the control group. The difference between the groups was − 0.43 mm (95% CI: − 0.80 to − 0.05 mm, *p* = 0.03), which was statistically significant (*p* = 0.03). Both groups demonstrated similar healthy peri-implant soft tissue values at the final follow-up.

**Conclusion:**

Although the current study is based on a small sample of participants, the findings suggest that the methodology of the PRF and the control group approach can both be used for bone augmentation with a similar outcome. A significant, but clinically irrelevant, higher peri-implant marginal bone level was registered in the PRF group than in the control group. Patients in both groups were highly satisfied with the treatment.

**Trial registration:**

ClinicalTrials.gov Identifier: NCT04350749. Registered 17 April 2020. Retrospectively registered.

## Background

Implant-supported single crowns are characterised by high long-term survival and few biological and technical complications, which typically includes peri-implant marginal bone loss, screw-loosening and fracture of veneering material complications [1–3]. To achieve a successful treatment outcome, the implants must be inserted in sufficient bone volume of an adequate quality to obtain primary stability enabling establishment of osseointegration [4, 5]. In many patients, this can be challenging due to extensive atrophy of the alveolar ridge after tooth loss [6] which compromises implant placement in a correct anatomical position [7, 8]. In cases where extensive reduction of the alveolar bone causes inability to achieve primary stability of the implant, the gold standard for lateral ridge augmentation involves an autogenous bone graft harvested from an intraoral donor site covered by a deproteinised bovine bone mineral (DBBM) and a resorbable collagen barrier membrane [9, 10]. The survival of implants placed in lateral augmented autogenous bone is high and comparable to that of implants placed in native bone [11–13]. However, the use of a barrier membrane may increase the risk of bone graft exposure due to soft tissue dehiscences, thereby compromising the success of the bone augmentation procedure [14]. Leukocyte and platelet-rich fibrin (PRF) is a platelet concentrate derived from a blood sample provided by the patient and produced without any anticoagulants [15, 16]. In vitro studies have demonstrated a positive effect of the use of PRF on cell proliferation, migration and adhesion in addition to anti-inflammatory and angiogenetic properties [17], which may have a beneficial clinical effect in bone augmentation procedures. Furthermore, the ability of PRF to inhibit osteoclastogenesis [18] may reduce bone resorption during the healing period. The PRF matrix can be compressed into a membrane, which has proven to be suitable as a scaffold for periosteal and osteoblastic tissue engineering [19, 20]. Despite the shape of a membrane, the PRF membrane does not have the properties of a resorbable barrier membrane [21, 22], due to its fast degradation in the same manner as a natural blood clot (1–2 weeks) [23]. Therefore, the PRF membrane is not believed to replace a barrier membrane in the classic understanding of guide bone regeneration (GBR), but rather to enhance the healing capacity of the periosteum and inwards to the augmented bone. Based on the positive effect of PRF in clinical and in vitro studies, it may therefore be speculated that adding PRF to a bone augmentation procedure may improve the vitality of the augmented bone, thereby causing accelerated bone remodeling [24]. In other words, dental implants can be inserted into more vital bone compared to the gold standard procedure, which from a clinical point of view is preferred to obtain optimal osseointegration [5]. In a recently published systematic review on clinical studies, it was concluded that PRF facilitates bone regeneration, although the evidence was moderate [25]. In oral and maxillofacial surgery, accelerated soft tissue healing has been demonstrated [23]. However, to our knowledge, no previous studies have presented the results of clinical and radiographic evaluation of implants placed in autogenous bone grafts covered by PRF membranes. Therefore, the purpose of this randomised, controlled clinical trial (RCT) was to compare the survival and clinical performance of implants placed in sites previously augmented with autogenous bone grafts covered by either a PRF membrane (PRF group) or a standard procedure (gold standard) involving coverage of the autogenous bone graft using a DBBM and a resorbable collagen membrane (control group). Our null hypothesis was that no difference between the test (PRF group) and the control group would exist.

## Material and methods

The study was performed according to the Declaration of Helsinki and internationally accepted guidelines for RCT, including the CONSORT statement (www.consort-statement.org).

The volumetric changes of the augmented bone [26], the histological composition of the augmented bone [27] and pain after the primary bone augmentation procedure [28] were previously described in detail.

### Patients

Eligible patients were randomly included (block randomisation with 20 patients in the first block and two patients in each of the following blocks) in a test (*n* = 14) and a control group (*n* = 13). Thus, a total of 27 consecutively treated patients (Table 1) were included according to the following inclusion criteria: (1) absence of one maxillary incisor, canine or premolar with indication for oral implant treatment, (2) severe atrophy of the alveolar process, classified as a type 2/4 defect by the “Classification of alveolar bone defects” by Terheyden [7] and hence potentially compromised primary stability with indication for lateral alveolar ridge augmentation before oral implant treatment, and (3) age > 20 years. The exclusion criteria were as follows: (1) systemic disease or medication compromising bone and soft tissue healing, (2) pathology in the edentulous region, (3) bruxism, (4) disease of the oral mucosa, (5) periodontal disease (probing depths ≥ 4 mm and a full mouth bleeding score ≥ 25%), (6) known allergies to bovine and porcine biomaterials and (7) no teeth adjacent the edentulous region.

**Table 1:** Demographics and survival rates of implants and implant crowns

	Test group (PRF)	Control group
Number of implants	14	13
Mean age, years (range)	47.9 (23–66)	52.3 (24–72)
Gender		
Female	6	6
Male	8	7
Smokers		
Total	2	1
< 10 cigarettes per day	1	
> 20 cigarettes per day	1	1
Number of implants	14	13
Implant length (mm) and implant diameter (mm)	L: 11.5, Ø: 3.75 = 2 L: 11.5, Ø: 4.3 = 2 L: 13, Ø: 3.75 = 5 L: 13, Ø: 4.3 = 5	L: 11.5, Ø: 3.75 = 4 L: 11.5, Ø: 4.3 = 0 L: 13, Ø: 3.75 = 6 L: 13, Ø: 4.3 = 3
Implant site, maxilla		
Incisors	3	6
Canine	1	
Premolars	10	7
Implant survival	100% (14/14)	85% (11/13)
Implant crown survival	100% (14/14)	92% (11/12)

*Abbreviations*: *L* length, *Ø* diameter

The same surgeon (JH) at the Section for Oral Surgery and Oral Pathology, Department of Dentistry, Health, Aarhus University, Denmark, performed all surgical procedures between 2015 and 2017. Three highly trained prosthodontists at the Section for Prosthetics, Department of Dentistry, Health, Aarhus University, performed the prosthodontic procedures between 2016 and 2018.

The mean age of the included patients at the time of inclusion was 48 years (range: 23–66 years) in the PRF group and 52 years (range: 24–72 years) in the control group (Table 1). At the time of the bone augmentation procedure, two patients (14%) in the PRF group and one (8%) patient in the control group were smokers. Patients were partially edentulous due to trauma (*n* = 22), agenesis (*n* = 3) or marginal periodontitis [2]. Two patients were unavailable for the final follow-up. The referring dentist followed the non-attenders, and telephone interview revealed no subjective or objective complications of either the implants or the crowns.

### Bone augmentation procedure

In the PRF group, the PRF membranes were prepared before surgery. A venous blood sample (80 ml, distributed in eight 10-ml glass-coated plastic tubes) was collected via puncture of a vein in the cubitial fossa and centrifuged using a Duo Quattro centrifugation device with a 40° rotor angulation with a radius of 88 mm at the clot and 110 mm at the max (A-PRF 12, Process, Nice, France) according to a previously described method [29–31]. We followed the manufacturer’s recommendations at the initiation of the study in 2015, producing the membranes using a protocol of 1300 RPM for 14 min (RCF-max = 208 g). The tubes containing the centrifuged blood were placed to rest for approximately 25 min to give the fibrin clot a firmer consistency before collecting it for the final membrane preparation, as previously recommended [31, 32]. The bone at the recipient and donor site were planned to be covered by three membranes, respectively (a total of six PRF membranes), while the last two tubules were held in reserve if for some reason clotting of the PRF matrix in the tubule was inadequate.

### Bone graft harvesting

**Fig. 1:**
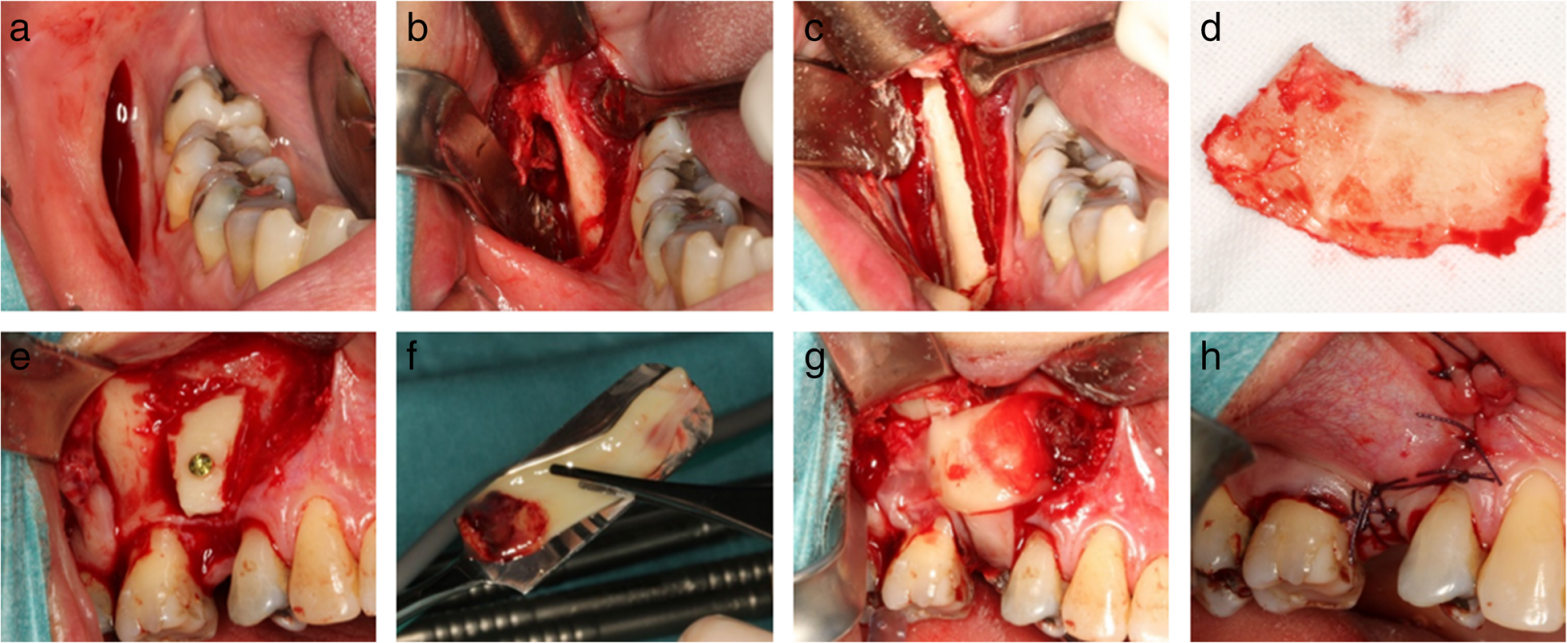
Intraoperative photos illustrating bone harvesting and lateral bone augmentation in the PRF group. Initially, an incision is made at the lateral aspect of the posterior part of the mandibular corpus (**a**) followed by exposing the mucoperiosteal flap (**b**), before making the osteotomy line (**c**). The bone block (**d**) is then retrieved before adjusted to the contour at the recipient site and fixated with an osteosynthesis screw (**e**). Autogenous bone graft particles are packed around the graft before covering the grafted area with three PRF membranes (**f** and **g**). Finally, tension-free primary wound closure is performed before suturing (**h**)

In local anaesthesia (Marcain®adrenalin, 5 mg/ml + 5 μg/ml, AstraZenca, Cambridge, UK) and via an intraoral approach, the lateral aspect of the posterior part of the mandibular corpus was exposed with a mucoperiosteal flap using a standard technique, as previously described [9, 14, 33] (Fig. 1a, b). The bone graft was retrieved by making a continuous osteotomy line with a cylindrical and a round bur at the lateral part of the mandible, with a uniform size of approximately 15 × 25 mm (Fig. 1c, d). The bone block containing mainly cortical bone was then gently separated from the mandible using a raspartorium. The block graft was covered with a saline-moistened gauze until used. In the PRF group, the donor site was covered by 3–4 PRF membranes while no adjunctive measures were performed in the control group [28], before suturing (4-0 Vicryl ^TM^, Ethicon ®, Johnson & Johnson, NJ, USA).

### Lateral bone augmentation

An incision was made on the top of the alveolar crest with 1–2 releasing incisions at the adjacent teeth before the mucoperiosteal flap was elevated. The previously collected bone block graft was adjusted to the contour of the bone at the recipient site and fixated with 1–2 osteosynthesis screws (Walter Lorenz® Midface System, Biomet Microfixation, Jacksonville, USA) (Fig. 1e). The remaining part of the autogenous bone graft was milled in a bone mill (Roswitha Quétin Dental-Produkte, Leimen, Germany), and autogenous bone graft particles were packed around the bone block. In the PRF group, three PRF membranes covered the grafted area (Fig. 1f, g). In the control group, the grafted area was covered by deproteinised bovine bone mineral (Geistlich Bio-Oss® Spongiosa Granules, Geistlich Pharma AG, Wolhusen, Switzerland) and two layers of a resorbable native bilayer collagen membrane (Geistlich Bio-Gide®, Geistlich Pharma AG, Wolhusen, Switzerland). Finally, the periosteum of the mucoperiosteal flap was released by an incision to secure tension-free primary wound closure before suturing (4-0 Vicryl ^TM^, Ethicon ®, Johnson & Johnson, NJ, USA) (Fig. 1h).

### Antibiotics, analgesic and plaque control

All patients received oral amoxicillin (1000 mg), metronidazole (500 mg), ibuprofen (400 mg), paracetamol (1000 mg) and methylprednisolone (32 mg) 1 h before surgery. Just prior to the operation, a mouth rinse with 10 ml 0.12% chlorhexidine digluconate was performed.

Methylprednisolone was prescribed the following morning (16 mg) and evening (16 mg). Additionally, postoperative ibuprofen (400 mg, four times daily) and paracetamol (1000 mg, four times daily) were prescribed for 1 week. The patients were instructed to rinse with 0.12% chlorhexidine digluconate twice daily and discontinue the use of their prostheses (if any). Patients were seen for consultation and suture removal 1–2 weeks postoperatively.

### Implant placement

Six months (mean 6.3 months, range: 4.8–7.8 months) after the initial bone augmentation procedure, all patients were recalled for implant installation. Prophylactic oral antibiotics (1000 mg amoxicillin, 1 h preoperatively) were given to all patients. A standard incision on the top of the alveolar process with one to two releasing incisions was completed before removal of the previously placed osteosynthesis screws. Using a trephine burr (Komet Dental, Lemgo, Germany, external diameter 3.2, internal diameter 2.6), we retrieved a cylindrical biopsy for later histological evaluation perpendicular to the lateral aspect of the augmented bone, approximately 10 mm from the top of the alveolar crest, including augmented bone and part of the native bone [27]. Finally, a submerged implant (NobelParallel Conical Connection, Nobel Biocare®, Zürich, Switzerland) was installed according to the manufacturer’s guidelines and using an implant surgical guide for optimal positioning. The implant top was positioned approximately 2.5 mm apically from the buccal gingival margin with an insertion torque of 35 Ncm. All patients were seen for consultation and suture removal 1 week postoperatively.

### Healing abutment operation

Approximately 7 months (mean 6.7 months, range: 5.7–10.0 months) after implant installation, all patients were recalled for healing abutment operation. A standard incision on the top of the alveolar process was completed before a healing abutment finally was placed after removal of the cover screw. Owing to the minimal incision, no suturing was necessary. The primary implant stability was determined by a percussion test and evaluation of an intraoral radiograph.

### Prosthodontic treatment

Forty-nine days (range: 27–113 days) after placement of the healing abutment, the abutment was removed and the implant position was registered by an impression coping on the implant. The final implant-supported restoration was fabricated by using an individually designed angulated screw channel (ASC) zirconium abutment (Nobel Biocare®, Zürich, Switzerland) and veneering porcelain. The metal adaptor (Nobel Biocare®, Zürich, Switzerland), ASC abutment (Nobel Biocare®, Zürich, Switzerland) and porcelain crown were screw-retained with a torque of 35 Ncm. All materials and clinical procedures were handled according to the manufacturer’s instructions.

### Follow-up regimen

All patients were recalled after a mean follow-up period of 24 months from crown placement (range: 14–32 months) by JH.

### Outcome measures

- Implant survival: Implant failure was defined as implant mobility or removal of a stable implant due to progressive peri-implant marginal bone loss or infection.
- Implant crown survival: Failure of the implant crown was defined as a loss of a mounted crown irrespective of the cause.
- Clinical parameters (probing depth, bleeding on probing, presence of plaque, keratinised peri-implant tissue, recession of peri-implant soft tissue)
- Radiographic peri-implant marginal bone change
- Radiographic marginal bone change of the adjacent tooth surfaces
- Biological and technical complication
- Patient-related outcome measures (PROMs)

Probing depth (PD) and bleeding on probing (BOP) were measured using a light probing force (approximately 25 g) to the nearest millimeter with a conventional periodontal probe at six sites per implant (mesiobuccal, buccal, distobuccal, distooral, lingual and mesiooral). Plaque was registered using a plaque control record (PCR; presence of plaque yes/no) [34]. Recession of peri-implant tissue (REC; six sites) and the width of the keratinised peri-implant tissue (KT; 1 buccal aspect of the implant) were measured to the nearest millimeter with the above-mentioned periodontal probe.

Intraoral radiographs were taken using the parallel technique at the time of implant installation, healing abutment operation and impression of the implant position as well as at the time of follow-up using a photostimulable phosphor system (Digora® fmx, Soredex Orion Cooperation, Helsinki, Finland) and stored as bmp files. The distance from the implant-abutment connection to the peri-implant marginal bone level was measured mesially and distally in parallel with the long axis of the implant using open-source software (ImageJ, National Institutes of Health, Bethesda, MD, USA). The distance from the cemento-enamel junction to the marginal bone level at the neighbouring tooth surfaces was also measured in parallel with the root surface [35]. The marginal bone level was defined as the most coronal level of the alveolar bone with a normal width of the periodontal ligament [36]. The correction of magnification was based on the known distance between the implant threads (0.6 mm) or implant length.

All patients were asked to fill out a questionnaire regarding their overall satisfaction with the implant treatment at the time of placement of the implant crown (baseline) and at the final follow-up. Their answers were registered using a 10-cm-long visual analogue scale (VAS) ranging from 0 (indicating discontent with the implant treatment) to 10 (indicating satisfaction with the implant treatment).

Each patient’s record was thoroughly reviewed, and all technical and biological complications during the follow-up period were registered. Two examiners (JH and FI) made all registrations and measurements.

### Data analysis

Data management and analysis including calculation of descriptive statistics were performed using Excel (Microsoft, Redmond, WA, USA) and STATA (StataCorp. 2019. *Stata Statistical Software: Release 16*. College Station, TX: StataCorp LLC, USA). No power calculation of sample size was included due to lack of relevant data on dental implants and platelet-rich fibrin in previously published studies. Data were analysed using a mixed model for repeated measurements. Comparisons within and between the groups were performed as post-hoc tests following the model. Normality of the residuals (the difference between the actual value of the outcome and the fitted values) and the homogeneity of the variance of the residuals were evaluated using the visual inspection of the QQ-plot of the residuals and a scatter plot of the residuals and the fitted values. The outcome of BOP and REC of the implant were dichotomised into absence or presence and analysed using a generalised linear model with log-link function analysing the ratio of the chance of BOP or REC (generally known as risk ratio). The remaining clinical parameters were tested using a simple linear regression model.

For interobserver repeatability, two observers (JH and FI) analysed the intraoral radiographs of five patients (20 radiographs). Additionally, for assessment of intrapersonal reproducibility, the images of all patients (104 radiographs) were measured twice (JH) allowing for a 3-month interval between the two measurements. The repeatability and reproducibility were described by the intraclass correlation coefficient (ICC) by a two-way mixed-effects model.

A statistically significant difference was considered when *p* < 0.05.

## Results

### Implant survival

Two of the 27 initially placed implants were lost in the control group (Table 1). Twenty months after placement of the implant-supported crown, one implant (first premolar, regular platform (4.3 mm), length: 13 mm) was lost due to failed osseointegration. No periodontitis or peri-implant marginal bone resorption was obvious at the time of implant removal. A second implant (central incisor, narrow platform (3.75 mm)) was lost during the placement of the final implant crown. For unknown reasons, a minimal rotation of the implant crown occurred several times when the abutment screw was torqued. In the phase of counter-torqueing the abutment screw, the implant loosened and was finally lost. Three months after the implants were lost, sufficient alveolar bone was still present in both patients and new implants were installed without further complications. Consequently, 11 out of 13 implants (85%, 95% CI: 62–104%) survived in the control group, and 14 out of 14 implants survived in the PRF group (100%). There was no statistical difference in implant survival between the groups (*p* = 0.13).

### Implant crown survival

None of the 26 initially placed implant crowns were lost, but one implant and therefore one implant crown was lost after 20 months. Consequently, the definitive implant crown survival was 92% (95% CI: 73–110%) in the control group and 100% in the PRF group. No statistical difference in implant crown survival was seen between the groups (*p* = 0.28).

### Probing depth

At the follow-up, the mean PD in the PRF group was 2.19 (95% CI: 1.95–2.43) mm at implant level with a variation of 1–4 mm at site level. In the control group, the mean PD was 2.13 (95% CI: 1.86–2.41) mm at implant level with a variation of 1–3 mm at site level. The difference between the groups was − 0.06 mm (95% CI: − 0.42–0.30). No statistical difference in PD was seen between the groups (*p* = 0.74).

### Bleeding on probing

The estimated probability or observed proportion of BOP for implants was 0.31 (95% CI: 0.14–0.70) in the PRF group and 0.30 (95% CI: 0.12–0.77) in the control group. The ratio of the probability of observing BOP was 1.046 (95% CI: 0.91–1.20), indicating that the probability of observing BOP is 4.6% higher in the PRF group than in the control group. No statistical difference in BOP was observed between the groups (*p* = 0.51).

### Plaque control record

The mean PCR in the control group was 8% (95% CI: − 2–19), whereas the mean PCR in the PRF group was 13% (95% CI: 4–22). The mean difference between the groups was 5% (95% CI: − 9–18%). There was no statistical difference in PCR between the groups (*p* = 0.51).

### Keratinised peri-implant tissue

The width of the keratinised tissue around the implant was 3.15 mm (95% CI: 2.30–4.01) in the PRF group and 3.40 mm (95% CI: 2.43–4.37) in the control group. The difference between the groups was 0.25 mm (95% CI: − 1.54–1.05). No statistical difference in the width of the keratinised peri-implant tissue was observed between the groups (*p* = 0.70).

### Recession of peri-implant soft tissue

The estimated probability or observed proportion of recession of > 0 mm buccal for implants was 0.15 (95% CI: 0.04–0.55) in the PRF group and 0.30 (95% CI: 0.12–0.77) in the control group. The ratio of the probability of observing recession of > 0 mm was 0.513 (95% CI: − 0.10–2.51), indicating that the probability of observing recession of > 0 mm was 4.87% lower in PRF group (1–0.513 = 0.487) than in the control group. No statistical difference in the recession of peri-implant tissue was observed between the groups (*p* = 0.41).

### Radiographic peri-implant marginal bone change

**Fig. 2:**
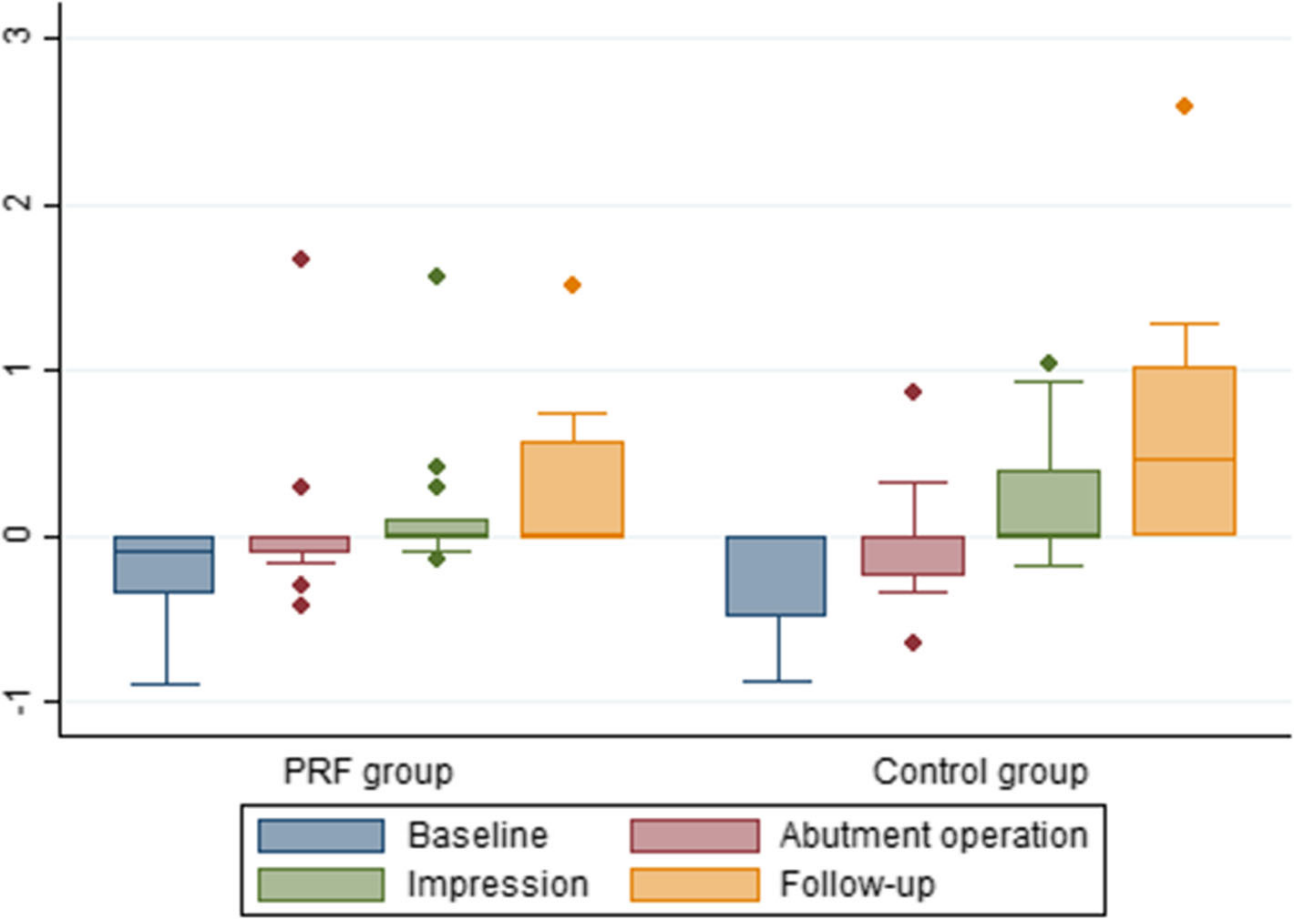
Box plot of the radiographic peri-implant marginal bone level at different time points in millimeter. Baseline: the time of implant placement; abutment: the time of abutment operation; impression: the time of impression taking; follow-up: the time of the final follow-up

The mean peri-implant marginal bone level at the different time points is shown in Table 2 and Fig. 2. The mean marginal bone level at follow-up was 0.26 mm (95% CI: 0.01–0.50 mm) in the PRF group and 0.68 mm (95% CI: 0.41–0.96 mm) in the control group. The difference between the groups was − 0.43 mm (95% CI: − 0.80 to − 0.05 mm, *p* = 0.03), which was statistically significant (*p* = 0.03). The peri-implant marginal bone level of the groups demonstrated the same progression over time (*p* = 0.0533).

**Table 2:** Radiographic peri-implant marginal bone level in mm

	Test group			Control group			Mean difference	95% CI	*p* value
	Obs	Mean	95% CI	Obs	Mean	95% CI			
Baseline	14	− 0.24	− 0.48 to 0.00	13	− 0.28	− 0.52 to 0.03	0.04	− 0.314 to 0.39	*p* = 0.82
Abutment	14	0.07	− 0.17 to 0.30	13	− 0.01	− 0.26 to 0.25	0.08	− 0.278 to 0.43	*p* = 0.66
Impression	14	0.15	− 0.09 to 0.39	13	0.20	− 0.05 to 0.44	− 0.05	− 0.40 to 0.30	*p* = 0.79
Follow-up	13	0.26	0.01 to 0.50	10	0.68	0.41 to 0.96	− 0.43	− 0.80 to − 0.05	*p* = 0.03

Baseline: the time of implant placement; abutment: the time of abutment operation; impression: the time of impression taking; follow-up: the time of follow-up. Negative values indicate that the implant-abutment connection was below the marginal bone level. The *p* values were calculated using post-hoc tests following the mixed model for repeated measurement

### Radiographic marginal bone change and soft tissue recession of adjacent tooth surfaces

From baseline to follow-up, the mean marginal bone loss was 0.14 mm (95% CI: 0.02–0.25 mm, *p* = 0.03) in the PRF group and 0.15 mm (95% CI: 0.04–0.26 mm, *p* = 0.01) in the control group (Table 3). This bone loss was statistically significant within the groups but not between the groups (*p* = 0.87). A minor soft tissue recession occurred on the adjacent teeth from baseline to follow-up. In the PRF group, a recession of 0.22 mm (95%: CI: − 0.62 to 0.19 mm, *p* = 0.26) was registered, while in the control group a recession of 0.07 mm (95% CI: − 0.74 to 0.60 mm, *p* = 0.83) was registered. No statistical difference within or between the groups was seen.

**Table 3:** Radiographic marginal bone level and clinical recession on neighbouring tooth surface

Group	Baseline (mean, 95% CI)	Follow-up (mean, 95% CI)	Difference (mean, 95% CI)	*p* value
Radiographic marginal bone level in mm				
Test	1.94 (1.50 to 2.38)	2.07 (1.64 to 2.51)	− 0.14 (− 0.25 to − 0.02)	*p* = 0.03
Control	2.34 (1.62 to 3.08)	2.49 (1.73 to 3.26)	− 0.15 (− 0.26 to − 0.04)	*p* = 0.01
			*p* = 0.87	
Recession in mm				
Test	0.88 (0.42 to 1.35)	1.10 (0.65 to 1.55)	− 0.22 (− 0.62 to 0.19)	*p* = 0.26
Control	1.17 (0.47 to 1.86)	1.23 (0.69 to 1.78)	− 0.07 (− 0.74 to 0.60)	*p* = 0.83
			*p* = 0.57	

Radiographic marginal bone level and clinical recession on neighbouring tooth surfaces at the time of baseline (before primary surgery) and at follow-up in the test and control group. The *p* values were calculated using the two sample *t* test with equal variance

### Complications

#### Primary augmentation

No dehiscence at the donor site was observed for any of the patients at the 1- and 2-week follow-up examination. One patient (control group) demonstrated bone graft dehiscence at the recipient site at both the 1- and 2-week follow-up. Although the block was reduced in thickness with a bur after it was exposed, soft tissue coverage was never obtained. Finally, the graft was removed and a second bone augmentation operation was successfully performed, without further complications.

One patient (control group) expressed minimally changed extraoral sensation in the chin region at both the 1- and 2-week follow-up. However, the extra- and intraoral clinical examination revealed no sensory disturbances. The patient was not affected by this and described the same changed sensation at the final clinical follow-up after 29 months.

Another patient (PRF group) experienced sensory disturbances at the mucosa of the alveolar sulcus at the recipient site, which was confirmed clinically. The disturbance decreased over time, but the patient was still affected by this at the final clinical follow-up 28 months later.

#### Implant placement

All implants could be placed 6 months after the bone block augmentation procedure. Buccal bone thickness after implant installation was less than 2 mm in two patients (one in each group); therefore, additional localised alveolar ridge augmentation was performed using locally harvested autogenous bone chips (Safescraper, Divisione Medical Meta, Italy) covered by Geistlich Bio-Oss® and a Geistlich Bio-Gide® membrane.

#### Technical and biological complications following implant placement

All patients were included in a maintenance care programme after placement of the implant crown by the referring dentist. No technical or biological complication was reported during the follow-up period by the referring dentist or at the final follow-up examination.

### Patient-reported outcome measures (PROMs)

**Fig. 3:**
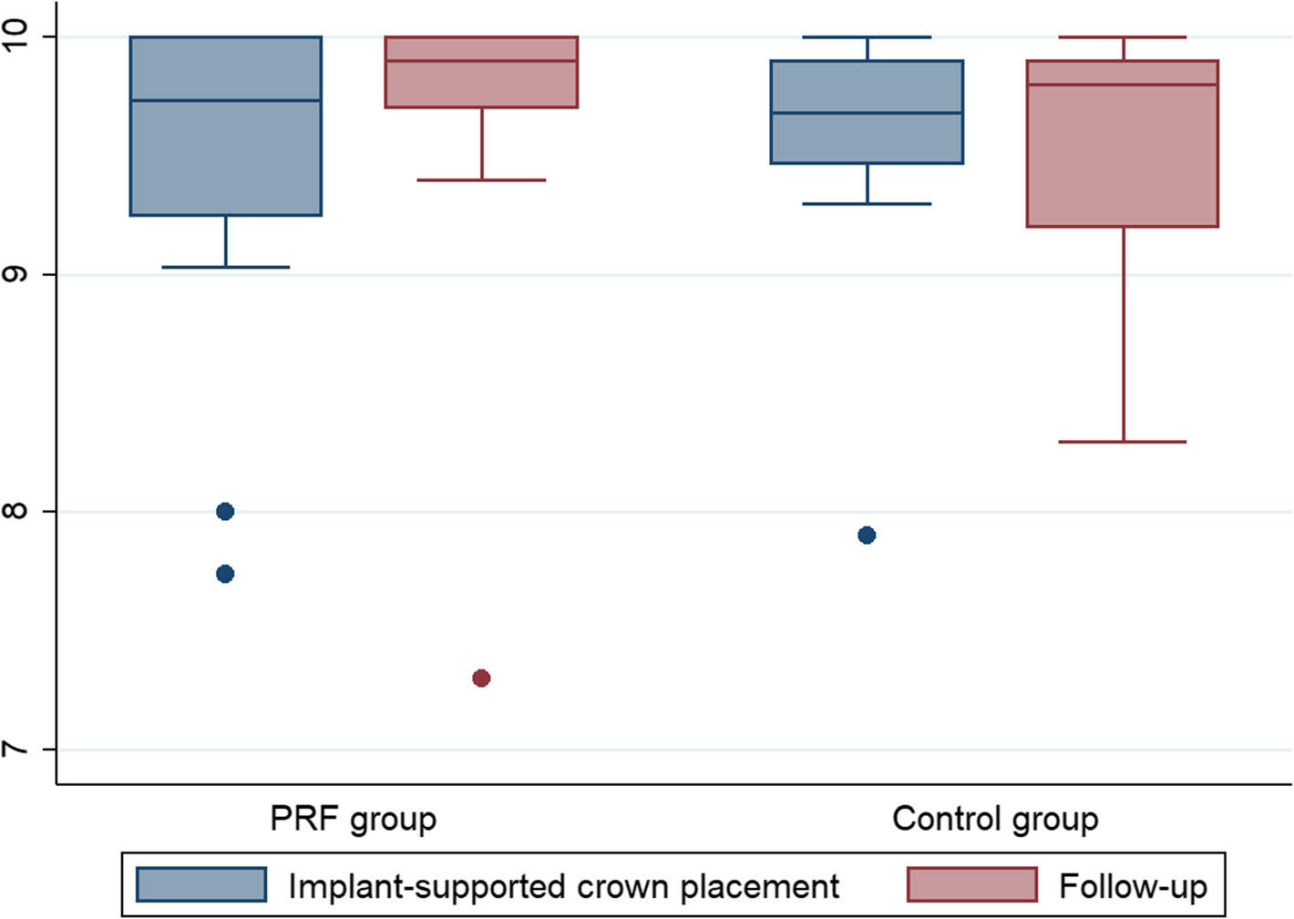
Data from the VAS of patient-related outcome measures at the time of mounting of the implant-supported crown and at the final follow-up of the PRF and control group

The overall treatment satisfaction was characterised as high at both the baseline examination and the follow-up examination for both groups (Table 4 and Fig. 3). At baseline, the mean PROM was 0.13 (95% CI: − 0.40 to 0.66, *p* = 0.61) higher in the control group than in the PRF group, while at the follow-up examination the control group was 0.10 (95% CI: − 0.66 to 0.46) lower than the PRF group. Within both groups, the mean PROM outcome was 0.22 (95% CI: − 0.19 to 0.63, *p* = 0.27) units higher at the follow-up examination than at the baseline examination in the PRF group and 0.02 (95% CI: − 0.48 to 0.44, *p* = 0.93) units lower for the control group. No statistical differences were observed between or within the groups. The change from baseline to follow-up examination was 0.24 (95% CI: − 0.37 to 0.85, *p* = 0.43) units higher for the PRF group than the control group.

**Table 4:** Patient-related outcome measures at baseline and at the final follow-up

	Test group	Control group	Difference	*p* value
	Mean (95% CI)	Mean (95% CI)	Mean (95% CI)	
Baseline	9.44 (9.09 to 9.78)	9.57 (9.20 to 9.95)	0.13 (− 0.40 to 0.66)	0.61
Follow-up	9.66 (9.30 to 10.02)	9.55 (9.15 to 9.96)	− 0.10 (-0.66 to 0.46)	0.71
Difference	0.22 (− 0.19 to 0.63)	− 0.02 (− 0.48 to 0.44)		
*p* value	0.27	0.93		

Data on the visual analogue scale of patient-related outcome measures at baseline (the time of placement of the implant-supported crown) and at the final follow-up. The *p* values were calculated using post-hoc tests following the mixed model for repeated measurement

### Reproducibility

The interobserver repeatability of the assessment of the radiographic peri-implant marginal bone level revealed a positive correlation between the two observers (*r*^*2*^ = 0.67, *p* = 0.001). Furthermore, a strong correlation between the first and second evaluation of the radiographic peri-implant marginal bone level was also revealed (*r*^*2*^ = 0.76, *p* < 0.001).

## Discussion

The present study focused on clinical and radiographic characteristics of staged implants placed in autogenous bone grafts covered by either a PRF membrane (PRF group) or a standard procedure (gold standard) involving coverage of the autogenous bone graft using a deproteinised bovine bone mineral and a resorbable collagen membrane (control group).

The PRF group demonstrated a high implant survival; however, two out of 13 implants were lost in the control group. The difference between the groups was non-significant, although implant survival in the control group to some extent differs from that reported in previous studies on survival of implants placed in bone grafts [12, 13]. One implant (first premolar, regular platform (4.3 mm), length: 13 mm) and the corresponding implant-supported crown were lost 20 months after final crown placement due to failed osseointegration. The reason for the implant loss remains unclear since no preceding biological complications were reported. The fact that the biopsy was taken from a relatively narrow bone block may have compromised the clinical outcome of the implant treatment, although none of the bone blocks were clinically loosened during the biopsy procedure. Harvesting of bone biopsies of larger autogenous bone blocks followed by implant placement has previously been described [37, 38], but no follow-up on implant survival has been reported. Another possible explanation for the loss of the second implant (central incisor, narrow platform (3.75 mm)) were problems with rotation of the ASC abutment in relation to the metal adaptor when tightening the abutment screw at the time of placement of the final crown. While counter-torqueing the abutment screw, the implant loosened and was finally lost. Only original components were used when fabricating the implant-supported crown, and the reason for the minimal rotation remains unclear. The combination of the NobelParallel CC implant launched in 2015 and an abutment with ASC is relatively new and has so far been lined to only few mechanical problems [39, 40], among which rotation of the crown when torqueing the abutment screw was not stated. In both patients, a new implant was placed without any need for additional bone augmentation and without further complications. Apart from these biological and mechanical problems, no additional complications were registered.

Assessment of PD and BOP revealed that most implants were characterised by healthy peri-implant tissues. The mean PD at implant level was 2.19 mm in the PRF group and 2.13 mm in the control group. Similar findings of long-term clinical outcomes of implants placed in an autogenous bone block [12] and native bone have previously been published [41]. All implants were positioned approximately 2.5 mm apically from the buccal gingival margin, which in some patients resulted in placement of the implant top apically to the marginal bone level (Table 2, Fig. 2). At the final follow-up, both groups demonstrated favourable peri-implant marginal bone levels, although the PRF group revealed a 0.43 mm (*p* = 0.03) higher peri-implant marginal bone level than the control group, meaning that the bone level was higher around the implant for the test group. This difference may be caused by a higher number of incisor implants in the control group (Table 1), since a more pronounced bone resorption rate in the anterior region compared to the posterior region following bone block augmentation has previously been described [26]. One patient in the PRF group demonstrated bone resorption around the implant at the abutment operation of more than 1 mm, but the marginal peri-implant bone level of that implant was stable both when the impression was taken and at the final follow-up. In contrast, one patient in the control group demonstrated a stable peri-implant marginal bone level both at the abutment operation and when the impression was taken, but showed a peri-implant marginal bone loss of more than 2 mm at the final follow-up. Consequently, the long-term prognosis of this implant may be compromised. The histological evaluation [27] of the biopsies retrieved at implant placement of this patient sample has previously been described. The above-mentioned patients were the only two patients (2/25 patient) in which their bone biopsy was characterized by moderate to heavy inflammation, indicating that the bone resorption and thereby the peri-implant marginal bone level are associated with resolution of the inflammatory process [42]. For both groups, the peri-implant marginal bone level of the implants was comparable to levels reported in previous studies involving implant placement in non-augmented and augmented sites [12, 13, 39].

A minor, but statistically significant, radiographic bone loss occurred from baseline to the final follow-up at the neighbouring tooth surfaces in both groups. Moreover, both groups experienced a minor recession of the marginal gingiva from baseline to the final follow-up, but the change was not significant. Recession and the bone level of the neighbouring tooth surfaces to implants placed in autogenous bone grafts have not been assessed previously, but preservation of the marginal bone level of the neighbouring tooth surfaces is important to preserve the vertical position of the papillae [43]. Obviously, recession around the teeth has a clinically and aesthetically adverse effect [44].

Some complications were registered in the process from the primary bone augmentation to implant placement, including a loss of one bone block and a minor change of intra- and extraoral sensitivity. Also, at the time of implant placement, simultaneous bone augmentation was necessary due to bone resorption of the primary augmented bone block in two patients (one patient in each group). This finding is consistent with previously described complications after bone block augmentation [10, 45], and in both patients, no further complications were registered. Despite these observed complications, the rating of the patient questionnaire revealed an overall high satisfaction with treatment at baseline and at the follow-up.

The prospective study design involving randomisation as well as a standardised surgical technique and systematic postoperative follow-up is an important strength of this study. Some weaknesses should also be acknowledged. First, it is important to bear in mind the potential bias associated with taking a biopsy from a relatively narrow bone block on the long-term results for implant treatment. It is possible that the clinical result of losing two implants is associated with the mechanical force applied on the bone block when retrieving the bone biopsy. This should be considered in future scientific work involving bone biopsy from a narrow bone block. Another limitation of the present study is the small sample of participants and the distribution of different recipient sites. The results should therefore be interpreted with caution.

## Conclusion

Although the current study is based on a small sample of participants, the findings suggest that the methodology of the PRF and the control group approach can both be used for bone augmentation with a similar outcome. A significant, but clinically irrelevant, higher peri-implant marginal bone level was registered in the PRF group than in the control group. Patients in both groups were highly satisfied with the treatment.

## Data Availability

The datasets used and analysed during the present study are available from the corresponding author on reasonable request.

## Abbreviations

ASC: Angulated screw channel
BOP: Bleeding on probing
DBBM: Deproteinised bovine bone mineral
GBR: Guided bone regeneration
ICC: Intraclass correlation coefficient
KT: Keratinised peri-implant tissue
PCR: Plaque control record
PD: Probing depth
PRF: Platelet-rich fibrin
PROM: Patient-related outcome measures
RCF: Relative centrifugal force
RCT: Randomised controlled trial
REC: Recession of peri-implant tissue
VAS: Visual analogue scale
